# Oncolytic reovirus as a combined antiviral and anti-tumour agent for the treatment of liver cancer

**DOI:** 10.1136/gutjnl-2016-312009

**Published:** 2016-11-15

**Authors:** Adel Samson, Matthew J Bentham, Karen Scott, Gerard Nuovo, Abigail Bloy, Elizabeth Appleton, Robert A Adair, Rajiv Dave, Adam Peckham-Cooper, Giles Toogood, Seishi Nagamori, Matthew Coffey, Richard Vile, Kevin Harrington, Peter Selby, Fiona Errington-Mais, Alan Melcher, Stephen Griffin

**Affiliations:** 1 Leeds Institute of Cancer & Pathology (LICAP) and Leeds Cancer Research UK Clinical Centre, Faculty of Medicine and Health, University of Leeds, St James’ University Hospital, Leeds, UK; 2 The Ohio State University, Comprehensive Cancer Centre, Columbus, Ohio, USA; 3 Department of Virology II, National Institute of Infectious Diseases 1-23-1 Toyama, Tokyo, Japan; 4 Oncolytics Biotech, Calgary, Alberta, Canada; 5 Department of Immunology, Mayo Clinic, Rochester, Minnesota, USA; 6 Department of Molecular Medicine, The Institute of Cancer Research, London, UK

**Keywords:** IMMUNOTHERAPY, HEPATITIS C, HEPATOCELLULAR CARCINOMA, ANTIVIRAL THERAPY, CANCER IMMUNOBIOLOGY

## Abstract

**Objective:**

Oncolytic viruses (OVs) represent promising, proinflammatory cancer treatments. Here, we explored whether OV-induced innate immune responses could simultaneously inhibit HCV while suppressing hepatocellular carcinoma (HCC). Furthermore, we extended this exemplar to other models of virus-associated cancer.

**Design and results:**

Clinical grade oncolytic orthoreovirus (Reo) elicited innate immune activation within primary human liver tissue in the absence of cytotoxicity and independently of viral genome replication. As well as achieving therapy in preclinical models of HCC through the activation of innate degranulating immune cells, Reo-induced cytokine responses efficiently suppressed HCV replication both in vitro and in vivo. Furthermore, Reo-induced innate responses were also effective against models of HBV-associated HCC, as well as an alternative endogenous model of Epstein–Barr virus-associated lymphoma. Interestingly, Reo appeared superior to the majority of OVs in its ability to elicit innate inflammatory responses from primary liver tissue.

**Conclusions:**

We propose that Reo and other select proinflammatory OV may be used in the treatment of multiple cancers associated with oncogenic virus infections, simultaneously reducing both virus-associated oncogenic drive and tumour burden. In the case of HCV-associated HCC (HCV-HCC), Reo should be considered as an alternative agent to supplement and support current HCV-HCC therapies, particularly in those countries where access to new HCV antiviral treatments may be limited.

**Significance of this study**

**What is already known on this subject?**

- The majority of hepatocellular carcinomas (HCCs) are linked with an underlying oncogenic virus infection, namely HBV (54%) or HCV (31%), which drives oncogenesis via both indirect inflammatory and direct carcinogenic mechanisms. Accordingly, suppression of HBV/HCV infections may improve HCC clinical outcomes, but few patients with HCC worldwide are cured of their hepatitis infections due to cost, compliance and treatment toxicity issues.
- Therapy for patients with advanced HCC is limited; Sorafenib only improves median survival by 2–3 months, is associated with significant side effects and has not been recommended by the National Institute for Health and Care Excellence in the UK.
- Oncolytic viruses (OVs) show potential as cancer therapies, killing malignant cells by direct and immune-mediated mechanisms.

**What are the new findings?**

- Clinical grade oncolytic orthoreovirus (Reo) elicited interferon (IFN) secretion and innate immune activation within primary human liver tissue in the absence of cytotoxicity and independently of viral genome replication.
- Reo achieved therapy in cell line and murine models of hepatitis virus-positive/hepatitis virus-negative HCC and efficiently suppressed HCV replication in vitro through the effects of repressed replication in vitro, and in vivo, through the effects of type I IFNs.
- The antiviral effects of Reo were also applicable to in vitro endogenous models of HBV-HCC and Epstein–Barr virus-associated lymphoma. Of the other OVs tested, only measles virus Edmonston strain recapitulated the potent antiviral effects of Reo.

**How might it impact on clinical practice in the foreseeable future?**

- The deployment of select IFN-focused OVs in patients with cancer harbouring underlying oncogenic virus infections either as single agents or in combination therapy is anticipated to suppress pathogenic infections, improving organ health and extending cancer-specific survival.

## Introduction

Hepatocellular carcinoma (HCC) is among the highest causes of cancer-associated mortality and has limited therapeutic options. The majority of HCCs are linked with an underlying oncogenic virus infection, namely HBV (54%) or HCV (31%).^1^ HCV-associated HCC (HCV-HCC) has a particularly high risk of recurrence following surgical interventions, compared with either HBV-HCC or other HCC types.^2^ ^3^ Successful antiviral therapy prior to hepatic resection and/or liver transplant significantly reduces this risk,^4^ yet toxicity associated with conventional systemic interferon (IFN)-based HCV therapy has historically excluded many newly diagnosed patients with HCV-HCC from such treatment. New direct-acting antivirals (DAAs) with improved safety profiles are revolutionising HCV therapy, yet compliance issues are common,^5^ and the high cost of DAAs profoundly limits their use in poorer countries, where the prevalence of HCV-HCC is highest.

**Significance of this study**

- **How might it impact on clinical practice in the foreseeable future?**
- The deployment of select IFN-focused OVs in patients with cancer harbouring underlying oncogenic virus infections either as single agents or in combination therapy is anticipated to suppress pathogenic infections, improving organ health and extending cancer-specific survival.

For patients with advanced HCC, little is known regarding the impact of antiviral therapy upon existing tumours. Dogma attributes the emergence and progression of HCV-HCC to a bystander phenomenon, driven by chronic hepatic inflammation. However, growing evidence indicates that HCV is also directly oncogenic, further supporting the need for combined antiviral and anti-tumour therapy. Specifically, antiviral therapy improves patient outcomes in HCV-associated lymphoma;^6^ HCC is more common in HCV-positive livers compared with autoimmune hepatitis;^7^ HCV proteins are directly oncogenic in preclinical models^8^ and HCV-HCC is more likely to maintain expression of the tumour suppressor miRNA122, an essential HCV cofactor, highlighting a critical role for HCV in oncogenesis.^9^ Therapy for patients with advanced HCC is limited; Sorafenib only improves median survival by 2–3 months, is associated with significant side effects and has not been recommended by the National Institute for Health and Care Excellence in the UK.^10–12^ *Trans*-arterial chemotherapy is routinely used for patients with locally advanced disease/as a bridging therapy to transplant and has also been tested as a systemic treatment, but is frequently complicated by reactivation/exacerbation of underlying HCV/HBV infections.^13^ ^14^ Thus, given the poor prognosis for most patients with HCC, a combined antiviral and anti-tumour therapy might yield significant patient benefit.

Oncolytic viruses (OVs) show potential as cancer therapies; classically, tumour selective killing arises from direct cellular lysis expedited by virus-associated traits that favour replication within malignant cells. However, OVs are also potent inducers of host immunity, which is increasingly recognised to be the major component of their anti-tumour efficacy.^15^ Encouraging responses have been observed in late-stage clinical testing without significant toxicity, with the first OV recently licensed for the treatment of melanoma^16^ (‘T-Vec’, herpes simplex virus type 1 (HSV-1) encoding human Granulocyte macrophage colony stimulating factor (GM-CSF)). The liver and HCC have been the subject of several OV clinical studies,^17^ including JX-594, a modified vaccinia virus (VV) encoding GM-CSF,^18^ and our own investigations using oncolytic *Orthoreovirus* (Reo) in the treatment of metastatic colorectal cancer (CRC).^19^

We hypothesised that the proinflammatory nature of OV immunotherapy may exert concomitant benefit upon both the cancer and the underlying oncogenic virus infection through the stimulation of innate responses, namely IFNs. Accordingly, Reo-induced innate immune responses within primary liver tissue simultaneously effected tumour killing and suppression of HCV replication in vitro and in vivo. These responses did not require productive Reo replication and were applicable to other models of virus-associated tumours. We propose that this dual mode of action, combined with an excellent safety record, may favour the rapid deployment of OVs such as Reo for disadvantaged patients with advanced, virus-associated HCC.

## Results

### Reo-induced innate immune responses occur in normal, as well as cancerous liver

**Figure 1:**
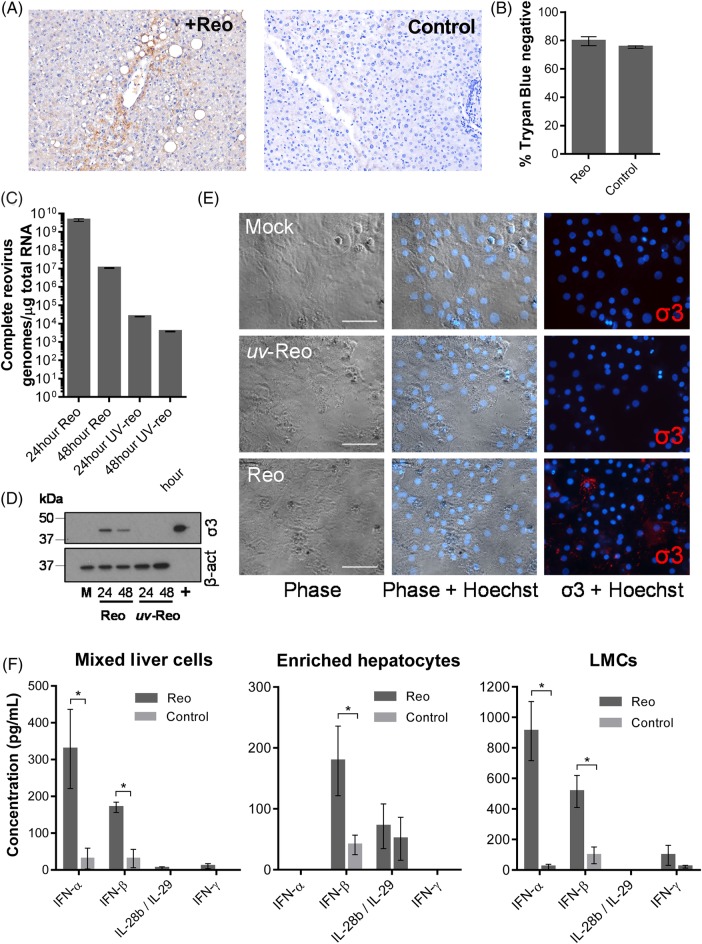
Reo reaches normal liver tissue following intravenous injection and stimulates interferon (IFN) secretion from ex vivo liver cells. (A) Immunohistochemistry (IHC) for Reo σ3 capsid protein (brown) from normal liver tissue derived from a patient treated intravenously with Reo (left) or an untreated control (right). Slides are representative of the clinical trial series or six untreated controls. (B) Viability assay for enriched ex vivo hepatocytes treated using Phosphate Buffered Saline (PBS) or 10 PFU/cell Reo for 72 hours and assayed for membrane integrity by Trypan Blue staining. (C) quantitative reverse transcriptase (qRT)-PCR for Reo σ3. Primary human hepatocytes were treated using 1 PFU/cell Reo or *uv-*Reo and incubated for 24 or 48 hours prior to RNA extraction. (D) Western blot for Reo σ3 and β-actin. Primary human hepatocytes were treated using 1 PFU/cell Reo or *uv-*Reo and incubated for 24 or 48 hours. Mock (M)-treated hepatocytes and RNA purified from Reolysin stocks (+) served as negative and positive controls. (E) Human hepatocytes were infected with Reo, or *uv*-Reo as above, and subjected to immunofluorescence analysis for Reo σ3 capsid protein, with Hoechst nuclear counterstain. (F) ELISA for IFN-α, IFN-β, interleukin (IL)-28b/IL-29 and IFN-γ derived from ex vivo mixed liver cells, enriched hepatocytes and liver mononuclear cells (LMCs) following stimulation with Reo or PBS control. * signifies p<0.005.

We previously confirmed that Reo efficiently targets CRC liver metastases following intravenous infusion, despite patient neutralising antibodies.^19^ Cell-mediated virus carriage enabled Reo replication within tumours and recovery of infectious virus from tumour explants. However, staining of surrounding normal liver tissue was also demonstrable in a number of patients, but infectious virus could not be isolated. This led us to re-examine normal liver patient tissue, confirming Reo protein expression within 5 out of 10 subjects (figure 1A).

Next, we examined Reo infection of normal ex vivo human liver tissue, generating single-cell suspensions. Mixed liver cells were fractionated into enriched hepatocyte and liver mononuclear cell (LMC) subpopulations (see online supplementary figure S1a). Importantly, exposure of enriched primary hepatocyte populations to Reo did not cause cellular toxicity (figure 1B), but did result in limited transcription of viral RNA (figure 1C) and protein expression 24 hours post-infection (figure 1D, E) in the absence of de novo infectious virion production (see online supplementary figure S1b). Interestingly, RNA and protein expression decreased between 24 and 48 hours, suggestive of the induction of cellular antiviral mechanisms (figure 1D, E). By contrast, Reo replicated efficiently within a broad range of HCC lines in vitro, producing new infectious virions (see online supplementary figure S1c), and inducing cytotoxicity (see online supplementary figure S1d), primarily via apoptosis (see online supplementary figure S1e). Importantly, potent cytokine responses were observed upon ex vivo exposure of mixed liver cells, hepatocyte-enriched or LMC fractions to Reo (figure 1F). All cell types expressed IFN-β, whereas IFN-α and IFN-γ segregated primarily within the LMC fraction, and IFN-λ (interleukin (IL)-28b/IL-29) was more prominent within hepatocyte-enriched fractions. Thus, Reo has the potential to stimulate innate immunity within a significant proportion of the liver, rather than being restricted to tumour cells and/or tumour-associated LMC.

10.1136/gutjnl-2016-312009.supp1 Supplementary data

### Reo-induced innate immune responses mediate anti-HCC therapy

**Figure 2:**
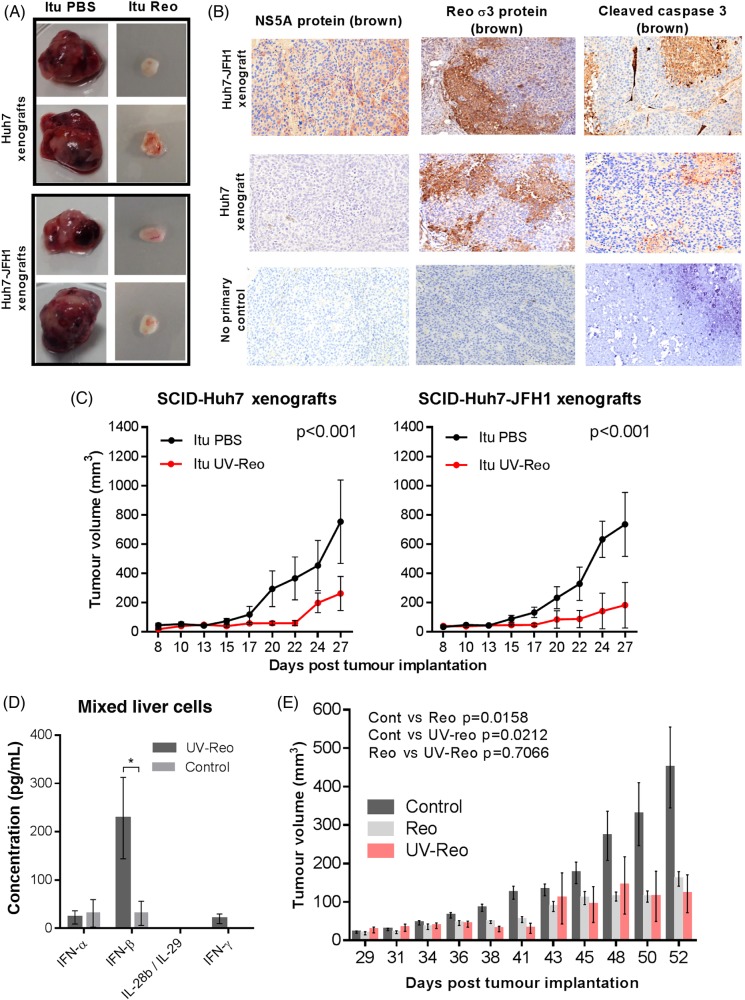
Reo-induced innate immune responses are sufficient for anti-hepatocellular carcinoma (HCC) therapy. (A) Excised subcutaneous flank tumours from SCID mice following a single Itu injection of PBS or 1×10^6^ PFU Reo. (B) Representative IHC staining (brown) for HCV non-structural protein (NS)5A, Reo σ3 capsid protein and cleaved caspase 3 in Huh7 and Huh7-JFH1 subcutaneous xenografts in SCID mice treated with a single Itu injection of 1×10^6^ PFU Reo on the 6th day post tumour implantation. (C) Tumour volume of Huh7 (left) and Huh7-JFH1 (right) subcutaneous flank xenografts in SCID mice treated with 3 times weekly Itu injections of PBS or 1×10^6^ PFU uv-Reo for 4 weeks starting the 6th day post tumour implantation. (D) ELISA for interferon (IFN)-α, IFN-β, IFN-γ and interleukin (IL)-28b/IL-29 derived from ex vivo mixed liver cells following stimulation with uv-Reo or PBS control. (E) Tumour growth of 1MEA subcutaneous syngeneic tumours in BALB/c mice treated with 3 times weekly Itu PBS, 1×10^6^ PFU Reo or 1×10^6^ PFU uv-Reo injections, starting the 6th day post tumour implantation. * signifies p<0.005.

To assess the efficacy of Reo against HCC models with/without HCV, we employed immunocompromised Severe combined immunodeficiency (SCID) mice bearing subcutaneous Huh7, or Huh7-JFH1 cell xenografts, carrying a subgenomic HCV replicon (genotype 2a, JFH-1 isolate). A single injection of Reo into palpable tumours (day 5 postimplantation) retarded tumour growth over a 3–4 week experiment, yielding small, macroscopically less vascular tumours (figure 2A). Histological examination of the tumours confirmed expression of HCV non-structural protein (NS)5A and association of Reo antigen with necrosis and cleaved caspase 3 (figure 2B). However, Reo-treated immunodeficient mice developed considerable toxicity evidenced by weight loss (see online supplementary figure S2a), necessitating premature sacrifice.

To circumvent toxicity issues and to delineate immune-driven, rather than lytic therapy, we revisited previous studies in melanoma models, where *uv*-inactivated Reo (*uv*-Reo) (see online supplementary figure S2b) primed antitumour immune responses comparably with live virus.^20^ Accordingly, *uv*-Reo caused significant retardation of both HCV-positive and HCV-negative tumour growth (figure 2C). *uv*-Reo also elicited innate cytokine responses from primary human mixed liver cell suspensions, although this was more focused to IFN-β (figure 2D). Finally, an immunocompetent HCC subcutaneous model (1MEA cells in syngeneic Balb/c mice) revealed similar reductions in tumour growth for Reo and *uv*-Reo, supporting an immune-driven, rather than an oncolytic mechanism of action (figure 2E). Taken together, Reo-induced innate immunity appears sufficient for anti-HCC effects in preclinical models, independent of Reo genomic replication, pointing to a major role for such a mechanism in humans undergoing treatment with live clinical grade virus.

### Reo-induced anti-HCC responses require IFN-mediated activation of natural killer lymphocytes

**Figure 3:**
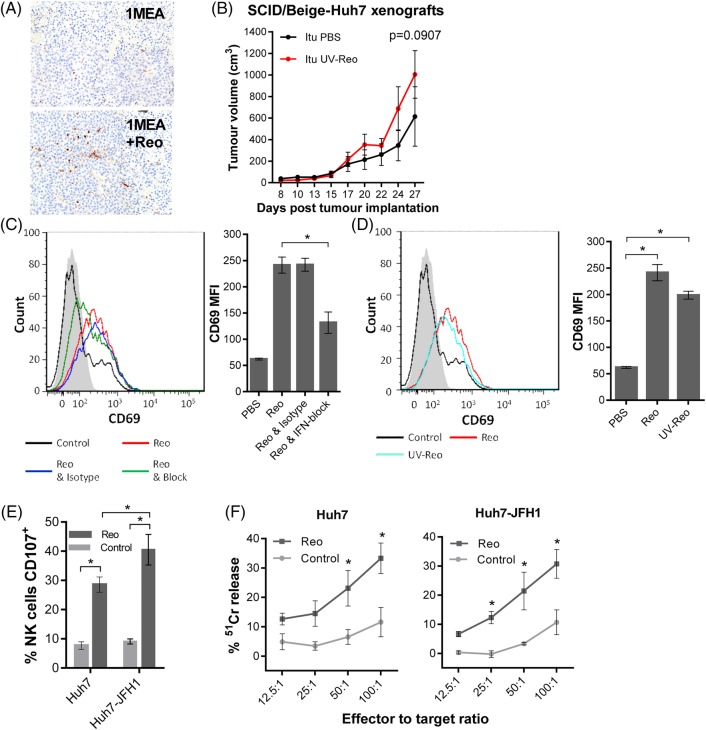
Reo-stimulated interferon (IFN) drives anti-hepatocellular carcinoma (HCC) immune responses via natural killer (NK) cell activation. (A) Representative IHC staining (brown) for NK cells in 1MEA subcutaneous syngeneic tumours in BALB/c mice treated with 3 times weekly Itu injections of PBS or 1×10^6^ PFU Reo. (B) Tumour growth of Huh7 subcutaneous xenografts in SCID/Beige mice treated with 3 times weekly Itu injections of PBS or 1×10^6^ PFU uv-Reo. (C) Flow cytometry overlay plot (left) and quantification (right) of median fluorescence intensity (MFI) for NK CD69 expression within liver mononuclear cells (LMCs) treated using PBS, 1 PFU/cell Reo, 1 PFU/cell Reo and type I IFN blocking antibodies or 1 PFU/cell Reo and isotype antibodies. (D) Similar to (C), but LMCs were treated with PBS, 1 PFU/cell Reo or 1 PFU/cell uv-Reo. (E) Degranulation assay showing per cent CD107-positive liver NK cells. LMCs were pretreated with PBS or 1 PFU/cell Reo for 24 hours, then coincubated with Huh7 or Huh7-JFH1 targets for 4 hours. (F) ^51^Cr release assay using LMCs pretreated with PBS or 1 PFU/cell Reo for 24 hours, then washed and coincubated with ^51^Cr-labelled Huh7 or Huh7-JFH1 targets for 4 hours. Data are ^51^Cr release as a percentage of the potential maximum. *signifies p<0.005.

Histological examination of Reo-treated 1MEA tumours revealed significant natural killer (NK) cell infiltration, indicating that these may represent an effector component of the antitumour response (figure 3A), as seen for other OVs.^21^ ^22^ Accordingly, *uv*-Reo treatment was ineffective against Huh7 xenografts within SCID/Beige mice that lack functional immune cell degranulation, consistent with a therapeutic requirement for NK cell activity (figure 3B). Interestingly, increased tumour growth was not observed in the SCID/Beige compared with the SCID context, suggesting that degranulating cells could not effectively control tumour growth in the absence of exogenous stimulation.

Consistent with murine models and our previous data, Reo effectively stimulated human liver-derived NK cells ex vivo via a type 1 IFN-dependent mechanism (figure 3C), thereby activating a major fraction within the LMC population (see online supplementary figure S1a). Likewise, *uv*-Reo stimulation of LMC also resulted in NK cell activation (figure 3D), as did Reo, also resulting in enhanced killing of both HCV-positive and HCV-negative Huh7 targets (figure 3E, F). Moreover, depleting NK cells from peripheral blood mononuclear cell (PBMC) prevented Reo-stimulated HCC killing (see online supplementary figure S3a), further supporting NK cells as major innate effectors in OV-mediated therapy. Thus, Reo-induced anti-HCC immunity is dependent upon type 1 IFN, which in turn activates NK cells, enabling them to recognise and kill tumour targets.

### Reo-induced immunity exerts potent anti-HCV effects in vitro

**Figure 4:**
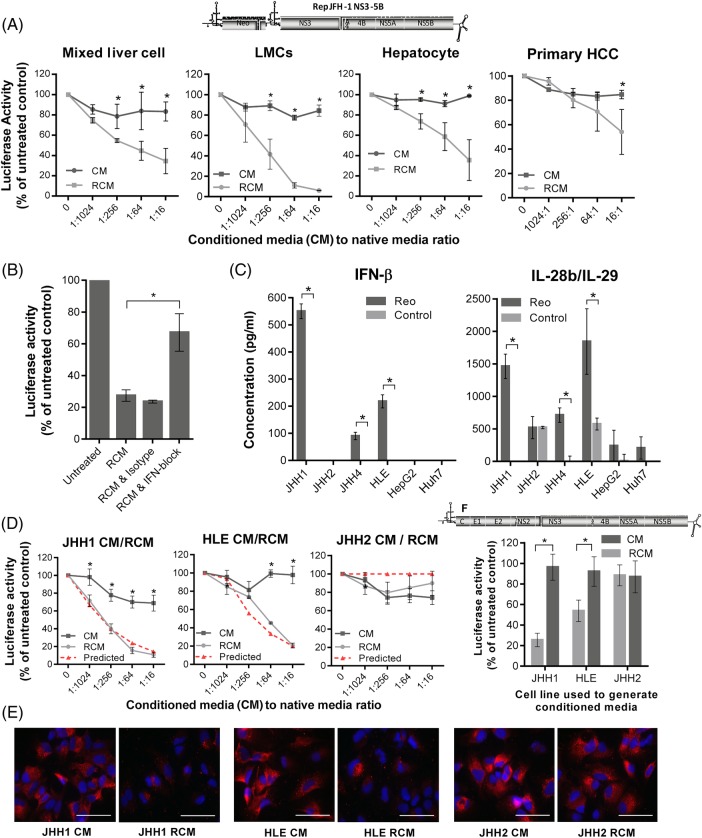
Reo-stimulated interferon (IFN)-β potently inhibits HCV in vitro. (A) Luciferase assay using Huh7-JFH1 cells treated for 24 hours with a range of dilutions of conditioned media (CM) or reovirus-CM (RCM) derived from mixed liver cells, liver mononuclear cells (LMCs), enriched hepatocytes or mixed primary hepatocellular carcinoma (HCC) cells. Luciferase activity was calculated as a percentage of control media values. (B) Luciferase assay using Huh7-JFH1 cells treated for 4 hours with mixed liver cell RCM (1:16 dilution), mixed liver cell RCM and type I IFN blocking antibodies or mixed liver cell RCM and isotype antibodies. Cells were then incubated in full growth media without treatment for a further 20 hours prior to analysis. (C) ELISA for IFN-β (left) and interleukin (IL)-28b/IL-29 (right) secretion from a panel of HCC cell lines following treatment for 72 hours with PBS or 1 PFU/cell Reo. (D) Luciferase assay using Huh7-JFH1 cells treated for 24 hours with a range of dilutions of CM or RCM derived from JHH1, HLE or JHH2 cells. Dotted red lines represent predicted per cent luciferase activity as a function of RCM IFN-β concentrations, as predicted from the trend line equation for replicon inhibition using purified IFN-β (see online supplementary figure 4SA). (E) Representative immunofluorescence for HCV non-structural protein (NS)5A in Huh7-JFH1 cells treated for 24 hours with CM or RCM at a dilution of 1:16 derived from JHH1, HLE or JHH2 cells. (F) Luciferase assays using HCV-infected Huh7 cells, treated for 24 hours with CM or RCM derived from JHH1, HLE or JHH2 cells at a dilution of 1:16. * signifies p<0.005.

Given the potent induction of inflammatory cytokines upon exposure of ex vivo human liver cells to Reo, we reasoned that resultant conditioned culture supernatants should exert antiviral effects. Filtered conditioned media (CM) were prepared following control, or Reo (reovirus-CM (RCM)) stimulation of mixed liver cells, fractionated hepatocyte-enriched and LMC populations or mixed primary HCC cells. All RCM exerted dose-dependent inhibitory effects upon HCV replication within Huh7 cells (figure 4A). Comparison with purified, leucocyte-derived IFN-α, IFN-β or IL-29, or combinations thereof, revealed a pattern consistent with a primarily IFN-β-mediated mechanism (see online supplementary figure S4a); IFN-β was significantly more potent in its ability to inhibit HCV replication compared with either IFN-α or IL-29, and cytokine combinations did not inhibit HCV replication more than IFN-β alone. IFN blockade significantly attenuated the HCV-inhibitory effect of mixed liver cell RCM (figure 4B), with no measurable cytotoxic or growth-inhibitory effects on Huh7-JFH1 cells (see online supplementary figure S4b).

Exposure of multiple HCC lines to Reo resulted in variable patterns of cytokine induction, lacking IFN-α, but with significant amounts of IFN-β and IFN-λ (figure 4C). We defined RCM derived from stimulated JHH1, HLE and JHH2 cells as retaining high, medium and low IFN-β content, respectively. Antiviral effects directly correlated with RCM IFN-β levels, mirroring the trend line equation for purified leukocyte-derived cytokine (Spearman's rank correlation of 0.929, p=0.001) (figure 4D, see online supplementary figure S4a). Concomitant decline in HCV protein expression was observed by both immunofluorescence (figure 4E) and western blot (see online supplementary figure S4c), again with no discernible cytotoxic effects (see online supplementary figure S4d). Critically, RCM antiviral activity was also applicable to full-length infectious HCV, again with direct correlation to IFN-β levels, measured by reductions in NS5A-luciferase reporter activity (figure 4F, see online supplementary figure S4e), HCV genomic RNA (see online supplementary figure S5a) and NS5A-Green Fluorescent Protein (GFP) reporter gene fluorescence (see online supplementary figure S5b, c).

### Reo-induced immunity eradicates HCV from cell culture and exerts antiviral effects in vivo

**Figure 5:**
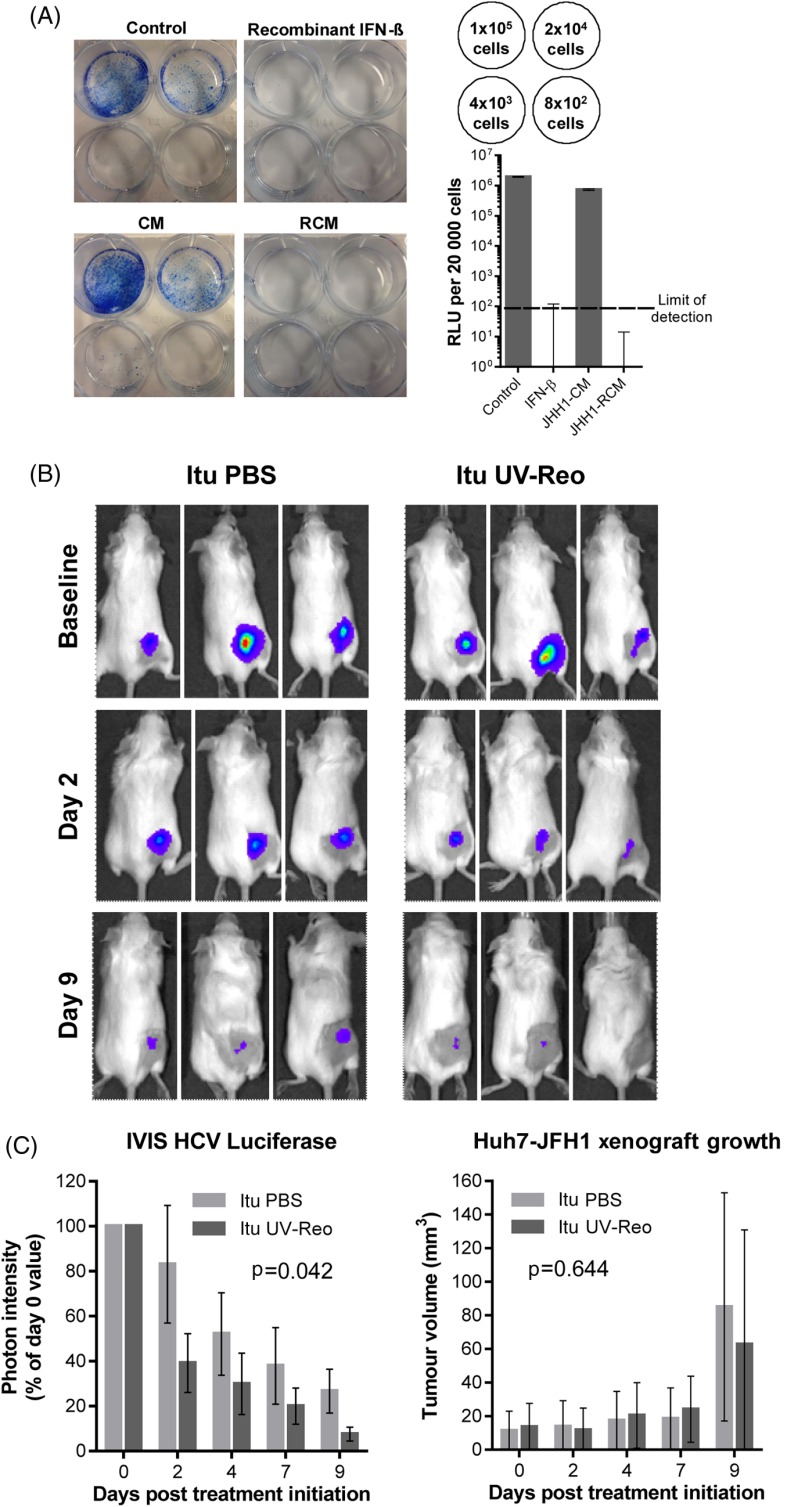
Reo-induced inflammatory responses eradicate HCV from cell culture and exert antiviral effects in vivo. (A) Huh7-JFH1 cells were treated for 14 days with reovirus-CM (RCM) or conditioned media (CM) derived from JHH1 cells at a dilution of 1:16, or with control media, or purified interferon (IFN)-β at 50 pg/mL. Cells were then either quantified for luciferase activity (bottom right) or seeded at the densities shown in top right and cultured in media containing G418 for 4 days before fixation and staining in methylene blue (left). (B) Representative in vivo imaging system (IVIS) pictures of HCV replicon luciferase activity within subcutaneous Huh7-JFH1 xenografts in SCID mice. Mice were treated with twice weekly Itu injections of PBS or 1×10^5^ PFU *uv*-Reo, starting immediately following baseline assessment. (C) (Left) Quantification of IVIS luciferase intensity from (B). Graph shows per cent luciferase activity in relation to day 0 post-treatment baseline values. (Right) Tumour volume of xenografts shown in (B).

We next examined whether RCM exposure could effectively cure Huh7-JFH-1 cells. Huh7-JFH-1 were exposed to RCM or control media for 14 days in the absence of selection. Rechallenge with G418 led to a lack of viable colonies in JHH1 RCM or IFN-β-treated cells, with undetectable luciferase activity in bulk populations (figure 5A).

Next, we tested whether *uv*-Reo-stimulated immunity could exert an antiviral effect in the preclinical SCID Huh7-JFH-1 model. Palpable subcutaneous Huh7-JFH-1 xenografts were treated intratumourally with either low-dose *uv*-Reo or PBS over a 9-day period, measuring tumour growth, as well as HCV expressed luciferase within tumours using an in vivo imaging system (IVIS). Tumours were normalised and segregated into equivalent control/treatment groups based upon IVIS readings at day 0. While luciferase readings naturally declined in controls due to the lack of G418, treatment with low-dose *uv*-Reo led to a significant additional reduction in IVIS readings, independent of tumour growth (figure 5B, C). Furthermore, histological examination of additional Huh7-JFH-1 tumours treated with a single injection of live Reo revealed marked reductions in HCV NS5A protein expression in areas of the tumour distinct from those undergoing necrotic changes or staining for Reo antigen, supportive of a *trans*-acting, cytokine-mediated antiviral effect (see online supplementary figure S6a). Accordingly, RCM generated from *uv*-Reo exposure of mixed murine liver cells retained anti-HCV activity in HUH7-JFH1 cells (see online supplementary figure S6b), albeit significantly reduced compared with human RCM. Hence, while murine RCM/IFN partially cross-stimulates Huh7 IFN receptors, murine xenograft models likely underestimate the magnitude of inhibition potentially achievable within a human context.

### Reo-induced antiviral responses are achievable in other models of virus-associated cancer

We reasoned that similar antiviral efficacy may be achieved in other models of virus-associated cancer. We assessed the effects of Reo-stimulated immunity against PLC/PRF/5 cells containing integrated HBV, and Daudi cells, which are derived from an Epstein–Barr virus (EBV)-positive Burkitt's lymphoma, known to be resistant to Reo-induced cytotoxic effects.^23^

**Figure 6:**
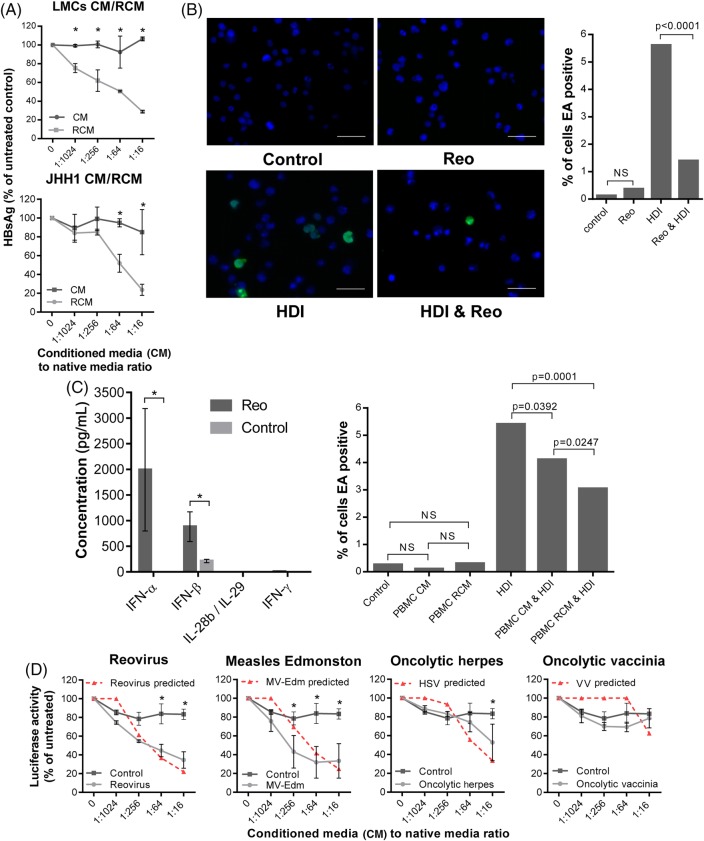
Reo-induced antiviral responses inhibit HBV and Epstein–Barr virus (EBV) in vitro. (A) ELISA for HBV surface antigen (HBsAg) secreted from PLC/PRF/5 cells treated for 5 days using a range of dilutions of conditioned media (CM) or reovirus-CM (RCM) derived from liver mononuclear cell (LMCs) (top) or JHH1 (bottom) cells. (B) Representative immunofluorescence (left) for EBV early antigen (EA) and quantification of EA-positive (right) Daudi cells. Cells were treated using PBS for 48 hours, 1 PFU/cell Reo for 48 hours, Histone deacetylase inhibitor (HDI) for 24 hours or 1 PFU/cell Reo for 48 hours followed by HDI for 24 hours. (C) (Left) ELISA for interferon (IFN)-α, IFN-β, IFN-γ and interleukin (IL)-28b/IL-29 derived from peripheral blood MC (PBMC) following stimulation with Reo or PBS control. (Right) Quantification of EA-positive Daudi cells treated for 24 hours using control media, PBMC CM, PBMC RCM, all followed by a further 24 hours treatment using either PBS or HDI. (D) Luciferase assay using Huh7-JFH1 cells treated for 24 hours with a range of dilutions of CM or 10 PFU/cell oncolytic virus (OV)-CM derived from mixed liver cells. * signifies p<0.005.

PLC/PRF/5 HBV surface antigen (HBsAg) secretion serves as a marker of viral gene expression.^24^ RCM derived from both primary LMC and also JHH-1 cells achieved reductions of HBsAg secretion comparable with purified IFN-β over a 5-day time course (figure 6A, see online supplementary figure S7a), with no observable growth-inhibitory effects (see online supplementary figure S7b). We next tested the ability of Reo to suppress EBV reactivation from tetradecanoylphorbol-13-acetate (TPA)/butyrate-treated Daudi cells. Both direct Reo treatment of Daudi cells and exposure to RCM derived from PBMC led to a significant reduction of EBV early antigen (EA)-positive cells (figure 6B, C). As Daudi cells are not permissive hosts, this effect was likely attributable to Reo-induced cytokines.

Finally, we assessed whether Reo was unique in its ability to generate potent anti-HCV cytokine responses from human primary mixed liver cells. From a panel of commonly researched OVs, only CM generated by exposure to measles virus Edmonston strain (MV-Edm) recapitulated the anti-HCV efficacy seen for Reo against Huh7-JFH-1 cells (figure 6D). Exposure to very high concentrations of HSV-1 CM generated only modest effects, whereas VV did not elicit appreciable antiviral responses. Moreover, correlation between antiviral effects and those predicted to be attributable to the induction of IFN-β (see online supplementary figure S7c) was significant (p<0.05) for all OVs except VV; low levels of cytokine were present in VV CM at similar concentrations to that released from mock-treated hepatic cells, yet neither exerted antiviral effects upon Huh7-JFH-1 cells. Hence, other factors present in control media may ameliorate the effects of low-level IFN or these low levels may not tally with the predictive equation (see online supplementary figure S7c). Taken together, Reo appears capable of inducing potent, IFN-β-driven antiviral responses that are effective against both RNA and DNA tumour viruses, yet this ability is not ubiquitous among OVs.

## Discussion

Here, we have provided the first evidence that OV-induced innate inflammatory responses simultaneously target oncogenic virus infections as well as associated tumours using primarily the exemplar of HCV-HCC, but with additional proof-of-principle for other models. Induction of type I IFN was a key, unifying mechanism determining both antiviral as well as anti-tumour efficacy in our model systems, and efficacy achieved using *uv*-Reo argues against a significant role for direct tumour targeting/lysis. Moreover, such qualities were not ubiquitous among OVs, making the selection of agent for future applications an important consideration.

Reo-induced IFN both exerted direct antiviral effects upon virus replication in vitro and anti-tumour effects in vivo via activation of degranulating innate immune cells, most likely NK cells. Hence, SCID/beige mice were unable to respond to *uv-*Reo therapy, yet this was not compromised by the lack of adaptive immunity within the SCID background. NK cells are indirectly activated by IFN,^25^ and they likely also contribute to in vivo antiviral effects via the killing of infected tumour cells/hepatocytes.^26^

IFN-β-mediated antiviral and anti-tumour effects in preclinical models were not dependent upon Reo replication, as *uv*-Reo primed similar responses from human hepatic cells. This is reminiscent of other OV studies, where the ability to complete a full infectious cycle is dispensable for the priming of IFN-mediated anti-tumour immunity.^20^ ^22^ ^27^ Nevertheless, it is likely that replication-competent virus provides other clinical advantages when treating preimmune human patients, such as potential amplification at tumour sites,^19^ and cell-mediated carriage^28^ to mitigate existing antibody responses; whether the latter occurs for *uv*-inactivated virus is unknown, and this would currently be impossible to test in vivo given the absence of clinical grade reagent. dsRNA human *Orthoreovirus* genomes are recognised by pattern recognition receptors, including RIG-I,^29^ although whether this is also true for *uv*-Reo is unclear. Other reoviruses, such as Carp reovirus, are also known to trigger responses through Toll-like-receptor pathways,^30^ yet the extent to which these are activated by *uv-*Reo in human liver cells is unknown. We cannot rule out a contribution of adaptive immunity to therapy in syngeneic 1MEA models, but this would likely be in addition to the essential innate anti-tumour response.

This study provides a novel antiviral dimension to OV-induced innate responses, which in turn also mediate much of their anti-tumour efficacy. This could have significant benefit for cancers such as HCC, for which therapeutic options are limited and the majority occur as a result of either HBV or HCV infection. Preclinical models available for the study of HCV or indeed HBV infection/replication remain severely limited, and all are set within altered genetic or induced immunocompromised backgrounds.^31^ ^32^ Furthermore, transgenic murine systems that spontaneously develop HCC in an HCV context necessarily express viral proteins under autologous promoters rather than in a replicative context, making these unsuitable for testing OV-mediated therapy. The Huh7-JFH1 xenograft models employed in the present study are also limited, but provide the advantage of replicating HCV within an HCC context, where responses to innate immune signalling remain intact.

New DAAs targeting HCV have recently been shown to be well tolerated in patients with HCV-HCC.^33^ Interestingly, sustained DAA responses in patients with chronic HCV were recently shown to correlate with restoration of hepatic type I IFN signalling homeostasis and increased expression of a defined series of IFN-stimulated genes (ISGs) upon the cessation of treatment.^34^ OV treatment combined with DAAs could conceivably improve the rate at which such intrahepatic ISG expression takes place, effectively improving viral clearance in patients with HCC. However, the likelihood is that the majority of patients with access to DAAs will have their HCV infection cured prior to the development of HCC, and the accumulating evidence that HCV is directly oncogenic provides a strong rationale to adopt the same practice for patients with advanced HCC. Thus, Reo could have particular relevance in countries unable to support widespread administration of DAAs, where both HCV and so also HCC prevalence may be higher. Furthermore, Reo anti-HCC effects could conceivably support or serve as second-line therapy to Sorafenib standard of care or form combinations with *trans*-arterial chemotherapy. This may become increasingly relevant in light of recent controversial studies supporting that DAA therapy may not be as effective as IFN in preventing the recurrence of HCV-HCC following surgery, although this awaits confirmation in much larger patient cohorts.^35^ ^36^

We have previously demonstrated that Reo-induced immunity is applicable to a number of varied tumour scenarios, and here we show that this innate immune activation displays antiviral efficacy. However, this trait was not common to all other OVs tested, with only MV-Edm sharing the ability of Reo to strongly induce type I IFN expression within primary human hepatic cells. Perhaps unsurprisingly, this suggests that OVs hailing from different virus families may vary in the principal mechanism by which they exert anti-tumour effects. Interestingly, VV, the platform for the modified JX-594, was a poor activator of innate responses within primary hepatic tissue; it is possible that the considerable array of innate immune antagonists encoded by VV contribute to this outcome.^37^ Interestingly, JX-594 therapy reduced HBV loads in a small cohort of patients with HCC in a trial substudy, yet this was not directly attributed to hepatic immune stimulation.^38^

The clinical potential for Reo to act as a combined antiviral and anti-tumour therapy in the context of HCV-HCC is supported by our finding that the virus can access normal liver tissue as well as tumours, following intravenous delivery in patients. Accordingly, exposure of ex vivo primary hepatic cells to Reo resulted in both viral RNA transcription and protein expression, although this did not culminate in the secretion of de novo infectious particles. This correlated with potent IFN induction within normal human liver cells in the absence of overt toxicity; hence, we infer that such responses limit, rather than prevent, viral replication within normal in vivo tissue, whereas this is less likely to occur in a tumour context, thereby subtly modifying the classical model for OV tumour specificity. As such, the favourable safety profile of Reo is well suited to the advanced hepatic disease state in patients with HCC, the majority of whom have underlying cirrhosis. Encouragingly, HCC biopsies from patients injected with OV in clinical trials showed diffuse lymphocyte infiltration,^18^ further supporting the use of OVs as combined antiviral and anti-HCC immunotherapies. Based upon their excellent safety records, we propose that the deployment of select OVs in patients with cancer harbouring underlying oncogenic virus infections be explored in clinical trials. Future research should combine IFN-focused OV such as Reo with other immunostimulatory agents, including immune checkpoint inhibitors (CI). Interestingly, the converse application of CI as antivirals is being explored for chronic infections such as HIV,^39^ suggesting that both Reo-like OVs and CI may form effective antiviral/anti-tumour combination therapies.

## Materials and methods

### Ethical standards

Ex vivo normal and malignant liver tissues were obtained from patients undergoing routine planned cancer-related surgery at St. James' University Hospital, Leeds, UK. Written informed consent was obtained in accordance with local institutional ethics review and approval.

### Immunohistochemistry

Employed an automated Bond Max system (Leica Biosystems) as described.^40^ Reovirus σ3 and cleaved caspase 3 antibodies were diluted to 1:1000. Detection of HCV NS5A included antigen retrieval and antibody dilution of 1:100. CD117 represented an NK cell-specific marker.^41^

### Fluorescence microscopy

Sheep anti-NS5A polyclonal serum (1:2000, from Mark Harris, Leeds),^42^ mouse anti-EBV EA-D clone (0261) (Santa Cruz, 1:100), Alexa Fluor-conjugated secondary antibodies (Invitrogen, 1:200) or direct GFP fluorescence was imaged using an EVOS FL Cell Imaging System or an Incuyte Zoom. 4F2 (reovirus σ3 antibody) was deposited to the DSHB by Dermody, T.S. (DSHB Hybridoma Product 4F2 (reovirus)). Huh7-JFH1 cells were treated with CM or RCM diluted 1:16 for 24 hours. Daudi cells were treated with 20 ng/mL TPA and 3 mM sodium butyrate.

### Preparation and quantification of HCV RNA

Total RNA was extracted from Huh7 cells containing HCV using TRIzol Reagent (Ambion), following the manufacturer's protocol. Reverse transcription was undertaken using a SensiFAST cDNA Synthesis Kit (Bioline). Quantification of HCV transcripts was undertaken using SYBR Green Real-Time PCR Master Mix (Applied Biosystems) on an Applied Biosystems 7500 Fast Real-Time PCR System, using standard conditions, with an annealing temperature of 63°C. The primer sequences were specific to the HCV 5′ UTR; Fwd, 5′-agcgtctagccatggcgt-3′ and Rev, 5′-ggtgtactcaccggttccg-3′, resulting in a 95 bp amplicon.

### Generation of (virus-)CM

CM and virus-CM (RCM) were derived from mixed liver cells or enriched hepatocytes treated with oncolytic reovirus, MV-Edm, VV or HSV-1 at 10 PFU/cell, or PBS, for 72 hours. Hepatoma lines, LMCs and PBMCs were treated with Reo at 1 PFU/cell for 72 hours. Supernatants were clarified at 400 ×g for 5 min, then filtered twice (2 µm (Corning) followed by OptiScale 25 Capsule (Millipore) to remove residual virus) and stored at −80°C.

### Type I IFN blockade

Huh7-JFH1 cells were treated with 1.25% (v/v) antihuman IFN-α/β receptor chain 2 or 1.25% (v/v) IgG2a isotype, R&D systems, and RCM is mixed with 0.75% (v/v) antihuman IFN-α and 0.75% (v/v) antihuman IFN-β, R&D systems or 1.5% (v/v) heat-inactivated sheep serum control, Sigma and incubated separately for 1 hour prior to mixing. After 4 hours, media was replaced without treatment. MTT/luciferase assays were conducted after a further 20 hours. LMCs were treated 1 hour prior to Reo stimulation and incubated for 24 hours prior to flow cytometry.

### Trypan Blue exclusion

Primary enriched hepatocytes were treated with PBS or 10 PFU/cell Reo for 72 hours, diluted 1:1 with 0.4% Trypan Blue (Sigma) and viability was determined from three replicates of 200 counted cells.

### Flow cytometry

Flow cytometry was performed using a BD LSRII flow cytometer and data were analysed on FACSDiva software (BD Biosciences). All antibodies comprised fluorescent-conjugated mouse monoclonal IgG1.

*Liver NK cell CD69*: washed LMCs were labelled with Fluorescein isothiocyanate (FITC)-CD56, PerCP-CD3 and PE-CD69 IgG1 antibodies (all BD Biosciences). Controls were labelled with PE-conjugated IgG1 isotype.

*CD107 a/b degranulation*: LMCs were incubated for 24 hours with 1 PFU/cell reovirus or PBS, washed twice in Hanks' Balanced Salt Solution (HBSS) (Sigma) and coincubated with target cells at a ratio of 5:1 for 4 hours. At 1 hour, cells were labelled with FITC-CD107a, FITC-CD107b antibodies (BD Biosciences), PE-CD56 (AbD Serotec) and PerCP-CD3 (BD Biosciences), with Brefeldin A solution (Biolegend).

### ELISA

IFN-α (Mabtech), IFN-β (Verikine Human IFN Beta ELISA Kit (PBL)), IFN-γ (BD Biosciences) and IL-28b/IL-29 (R&D Systems) were detected using matched HBsAg antibody pairs. PLC/PRF/5 cells were RCM treated for 5 days with daily replacement, followed by a final 3 hours treatment assessed for HBsAg using the Monolisa HBsAg ULTRA Kit (Bio-Rad), as per manufacturer instructions.

### Luciferase assay

Firefly (subgenomic HCV) or Renilla (infectious HCV) Luciferase assay systems (Promega) were assessed according to manufacturer's protocols using a MITHRAS luminometer (Berthold Technologie).

### ^51^Chromium release assay

^51^Chromium release was measured as described.^43^ Effector cells (LMCs or PBMCs) were preincubated for 24 hours with 1 PFU/cell of reovirus or PBS and dispensed in triplicate at the stated effector to target ratios.

### In vivo experiments

CB17-Prkdc*^SCID^* (SCID) mice were provided by the St. James' Biomedical Service (SBS). BALB/c mice and CB17 (Prkdc*^SCID^*, Lyst*^bg^* ‘SCID/Beige’) were purchased from Charles River Laboratories International. SCID and SCID/Beige mice were housed in isolator cages, while BALB/c mice were housed in individually ventilated cages. Cell lines were confirmed free of murine pathogens (Charles River Laboratories). Mice were regularly examined for signs of deterioration in health or weight loss.

Cell lines were harvested, washed twice in PBS and resuspended in 100 µL PBS for subcutaneous injection. All treatments were in 50 µL of vehicle fluid (PBS for Reo/*uv*-Reo or for Sorafenib by oral gavage in vehicle; PBS, 25% polyethylene glycol 400 (Sigma), 5% Tween-20, 5% ethanol).

Tumour growth was measured in two dimensions using callipers and mice were routinely sacrificed when 15 mm was reached in any dimension. Tumour volumes were calculated according to the modified ellipsoidal formula.^44^

For the IVIS (Caliper Life Sciences), SCID mice were anaesthetised by 1.5% isofluorane inhalation and injected intraperitoneally with 80 µL of 150 mg/mL Firefly D-luciferin potassium salt (Caliper Life Sciences) dissolved in PBS. Luciferase activity was measured after 10 min. Data were analysed using Living Image Software for IVIS (Perkin Elmer) by quantifying the total emitted luminescence within a circular area corresponding to the tumour injection site and subtracting the background emission.

### Statistics

p Values were calculated by the two-sided paired t-test for single points or groups, and statistical significance is denoted by *p<0.05. Cells or supernatants were assayed in triplicate when possible and data represent the mean and SEM between repeat experiments or samples from different donors.

## References

[1] Boyle P, Levin B World cancer report 2008. Lyon: International Agency for Research on Cancer, 2008.

[2] Franssen B, Alshebeeb K, Tabrizian P, et al Differences in surgical outcomes between hepatitis B- and hepatitis C-related hepatocellular carcinoma: a retrospective analysis of a single North American center. Ann Surg 2014;260:650–6; discussion 656–8 10.1097/SLA.0000000000000917 25203882

[3] Utsunomiya T, Shimada M, Kudo M, et al A comparison of the surgical outcomes among patients with HBV-positive, HCV-positive, and non-B non-C hepatocellular carcinoma: a nationwide study of 11,950 patients. Ann Surg 2015;261:513–20. 10.1097/SLA.0000000000000821 25072437

[4] Hsu CS, Chao YC, Lin HH, et al Systematic review: impact of interferon-based therapy on HCV-related hepatocellular carcinoma. Sci Rep 2015;5:9954 10.1038/srep09954 25963067 PMC4428066

[5] Hagan H, Campbell J, Thiede H, et al Self-reported hepatitis C virus antibody status and risk behavior in young injectors. Public Health Rep 2016;121:710–19. 10.1177/003335490612100611 PMC1781913 17278406

[6] Viswanatha DS, Dogan A Hepatitis C virus and lymphoma. J Clin Pathol 2007;60:1378–83. 10.1136/jcp.2007.051870 18042694 PMC2095565

[7] Teufel A, Weinmann A, Centner C, et al Hepatocellular carcinoma in patients with autoimmune hepatitis. World J Gastroenterol 2009;15:578–82. 19195059 10.3748/wjg.15.578 PMC2653348

[8] Wang AG, Lee DS, Moon HB, et al Non-structural 5A protein of hepatitis C virus induces a range of liver pathology in transgenic mice. J Pathol 2009;219:253–62. 10.1002/path.2592 19621337

[9] Bandiera S, Pfeffer S, Baumert TF, et al miR-122—a key factor and therapeutic target in liver disease. J Hepatol 2015;62:448–57. 10.1016/j.jhep.2014.10.004 25308172

[10] Llovet JM, Ricci S, Mazzaferro V, et al Sorafenib in advanced hepatocellular carcinoma. N Engl J Med 2008;359:378–90. 10.1056/NEJMoa0708857 18650514

[11] Cheng AL, Kang YK, Chen Z, et al Efficacy and safety of sorafenib in patients in the Asia-Pacific region with advanced hepatocellular carcinoma: a phase III randomised, double-blind, placebo-controlled trial. Lancet Oncol 2009;10:25–34. 10.1016/S1470-2045(08)70285-7 19095497

[12] NICE. Sorafenib for the treatment of advanced hepatocellular carcinoma | Guidance and guidelines | NICE. Published Online First: 2010 http://www.nice.org.uk/guidance/TA189 (accessed 6 Jul 2014).

[13] Lao XM, Luo G, Ye LT, et al Effects of antiviral therapy on hepatitis B virus reactivation and liver function after resection or chemoembolization for hepatocellular carcinoma. Liver Int 2013;33:595–604. 10.1111/liv.12112 23402625

[14] Mahale P, Kontoyiannis DP, Chemaly RF, et al Acute exacerbation and reactivation of chronic hepatitis C virus infection in cancer patients. J Hepatol 2012;57:1177–85. 10.1016/j.jhep.2012.07.031 22871500

[15] Melcher A, Parato K, Rooney CM, et al Thunder and lightning: immunotherapy and oncolytic viruses collide. Mol Ther 2011;19:1008–16. 10.1038/mt.2011.65 21505424 PMC3129809

[16] Andtbacka RHI, Kaufman HL, Collichio F, et al Talimogene laherparepvec improves durable response rate in patients with advanced melanoma. J Clin Oncol 2015;33:2780–8. 10.1200/JCO.2014.58.3377 26014293

[17] Jebar AH, Errington-Mais F, Vile RG, et al Progress in clinical oncolytic virus-based therapy for hepatocellular carcinoma. J Gen Virol 2015;96(Pt 7):1533–50. 10.1099/vir.0.000098 25711964

[18] Heo J, Reid T, Ruo L, et al Randomized dose-finding clinical trial of oncolytic immunotherapeutic vaccinia JX-594 in liver cancer. Nat Med 2013;19:329–36. 10.1038/nm.3089 23396206 PMC4268543

[19] Adair RA, Roulstone V, Scott KJ, et al Cell carriage, delivery, and selective replication of an oncolytic virus in tumor in patients. Sci Transl Med 2012;4:138ra77 10.1126/scitranslmed.3003578 PMC3893925 22700953

[20] Prestwich RJ, Ilett EJ, Errington F, et al Immune-mediated antitumor activity of reovirus is required for therapy and is independent of direct viral oncolysis and replication. Clin Cancer Res 2009;15:4374–81. 10.1158/1078-0432.CCR-09-0334 19509134 PMC4821072

[21] Prestwich RJ, Errington F, Steele LP, et al Reciprocal human dendritic cell-natural killer cell interactions induce antitumor activity following tumor cell infection by oncolytic reovirus. J Immunol 2009;183:4312–21. 10.4049/jimmunol.0901074 19734207

[22] Zhang J, Tai LH, Ilkow CS, et al Maraba MG1 virus enhances natural killer cell function via conventional dendritic cells to reduce postoperative metastatic disease. Mol Ther 2014;22:1320–32. 10.1038/mt.2014.60 24695102 PMC4088996

[23] Alain T, Hirasawa K, Pon KJ, et al Reovirus therapy of lymphoid malignancies. Blood 2002;100:4146–53. 10.1182/blood-2002-02-0503 12393565

[24] Yamashita Y, Koike K, Takaoki M, et al Suppression of HBsAg production in PLC/PRF/5 human hepatoma cell line by interferons. Microbiol Immunol 1988;32:1119–26. 10.1111/j.1348-0421.1988.tb01476.x 2464737

[25] Tu Z, Bozorgzadeh A, Pierce RH, et al TLR-dependent cross talk between human Kupffer cells and NK cells. J Exp Med 2008;205:233–44. 10.1084/jem.20072195 18195076 PMC2234385

[26] Krämer B, Körner C, Kebschull M, et al Natural killer p46 high expression defines a natural killer cell subset that is potentially involved in control of hepatitis C virus replication and modulation of liver fibrosis. Hepatology 2012;56:1201–13. 10.1002/hep.25804 22532190

[27] Wongthida P, Diaz RM, Galivo F, et al VSV oncolytic virotherapy in the B16 model depends upon intact MyD88 signaling. Mol Ther 2011;19:150–8. 10.1038/mt.2010.225 20959810 PMC3017452

[28] Ilett E, Kottke T, Donnelly O, et al Cytokine conditioning enhances systemic delivery and therapy of an oncolytic virus. Mol Ther 2014;22:1851–63. 10.1038/mt.2014.118 24957982 PMC4428400

[29] Loo YM, Fornek J, Crochet N, et al Distinct RIG-I and MDA5 signaling by RNA viruses in innate immunity. J Virol 2008;82:335–45. 10.1128/JVI.01080-07 17942531 PMC2224404

[30] Rao Y, Su J Insights into the antiviral immunity against grass carp (Ctenopharyngodon idella) reovirus (GCRV) in grass carp. J Immunol Res 2015;2015:670437 10.1155/2015/670437 25759845 PMC4337036

[31] Dorner M, Horwitz JA, Robbins JB, et al A genetically humanized mouse model for hepatitis C virus infection. Nature 2011;474:208–11. 10.1038/nature10168 21654804 PMC3159410

[32] Mercer DF, Schiller DE, Elliott JF, et al Hepatitis C virus replication in mice with chimeric human livers. Nat Med 2001;7:927–33. 10.1038/90968 11479625

[33] Curry MP, Forns X, Chung RT, et al Sofosbuvir and ribavirin prevent recurrence of HCV infection after liver transplantation: an open-label study. Gastroenterology 2015;148:100–7.e1. 10.1053/j.gastro.2014.09.023 25261839

[34] Meissner EG, Wu D, Osinusi A, et al Endogenous intrahepatic IFNs and association with IFN-free HCV treatment outcome. J Clin Invest 2014;124:3352–63. 10.1172/JCI75938 24983321 PMC4109554

[35] Reig M, Mariño Z, Perelló C, et al Unexpected high rate of early tumor recurrence in patients with HCV-related HCC undergoing interferon-free therapy. J Hepatol 2016;65:719–26. 10.1016/j.jhep.2016.04.008 27084592

[36] Conti F, Buonfiglioli F, Scuteri A, et al Early occurrence and recurrence of hepatocellular carcinoma in HCV-related cirrhosis treated with direct-acting antivirals. J Hepatol 2016;65:727–33. 10.1016/j.jhep.2016.06.015 27349488

[37] Smith GL, Benfield CTO, Maluquer de Motes C, et al Vaccinia virus immune evasion: mechanisms, virulence and immunogenicity. J Gen Virol 2013;94: 2367–92. 10.1099/vir.0.055921-0 23999164

[38] Liu TC, Hwang T, Park BH, et al The targeted oncolytic poxvirus JX-594 demonstrates antitumoral, antivascular, and anti-HBV activities in patients with hepatocellular carcinoma. Mol Ther 2008;16:1637–42. 10.1038/mt.2008.143 18628758

[39] Kaufmann DE, Walker BD PD-1 and CTLA-4 inhibitory cosignaling pathways in HIV infection and the potential for therapeutic intervention. J Immunol 2009;182:5891–7. 10.4049/jimmunol.0803771 19414738 PMC3726306

[40] Nuovo GJ, Garofalo M, Valeri N, et al Reovirus-associated reduction of microRNA-let-7d is related to the increased apoptotic death of cancer cells in clinical samples. Mod Pathol 2012;25:1333–44. 10.1038/modpathol.2012.95 22699519 PMC4275064

[41] Hughes T, Becknell B, McClory S, et al Stage 3 immature human natural killer cells found in secondary lymphoid tissue constitutively and selectively express the TH 17 cytokine interleukin-22. Blood 2009;113:4008–10. 10.1182/blood-2008-12-192443 19244159 PMC2673127

[42] Macdonald A, Crowder K, Street A, et al The hepatitis C virus non-structural NS5A protein inhibits activating protein-1 function by perturbing ras-ERK pathway signaling. J Biol Chem 2003;278:17775–84. 10.1074/jbc.M210900200 12621033

[43] Errington F, Jones J, Merrick A, et al Fusogenic membrane glycoprotein-mediated tumour cell fusion activates human dendritic cells for enhanced IL-12 production and T-cell priming. Gene Ther 2006;13:138–49. 10.1038/sj.gt.3302609 16136162

[44] Tomayko MM, Reynolds CP Determination of subcutaneous tumor size in athymic (nude) mice. Cancer Chemother Pharmacol 1989;24:148–54. 10.1007/BF00300234 2544306

