# Supplementary material for: Oncolytic reovirus as a combined antiviral and anti-tumour agent for the treatment of liver cancer
Source: Gut. 2016 Nov 15;67(3):562–73. doi: 10.1136/gutjnl-2016-312009 (PMC5868283; doi:10.1136/gutjnl-2016-312009)
Supplement: Supplementary data [file gutjnl-2016-312009supp001.pdf]

Supplementary Materials

Materials and Methods

Cell culture

Cells were cultured at 37 °C in humidified 5 % CO_2 and were routinely tested for *Mycoplasma.* JHH1, JHH2, JHH4, HLE and HepG2 were from the Japanese Collection of Research Biosources *Cell* Bank (JCRB). PLC/PRF/5, 1MEA, Vero and L929 were from the *American Type Culture Collection (*ATCC*).* Huh7.5 cells were provided by Jane McKeating, Birmingham. Media (with 10 % FCS and 1 % NEAA): JHH1, JHH2 William's Media E (Sigma); JHH4, PLC/PRF/5, Vero MEME (Sigma); HLE, HepG2, Huh7 (provided by John MacLauchlan, Glasgow CVR), Huh7.5, 1MEA, L929 DMEM (Sigma).

Huh7/7.5 cells were transfected with HCV replicon (Rep-Feo-JFH1), from Yutaka Amako and Aleem Siddiqui, (Boston, USA), or full-length J6/JFH-1-RLucΔ40 or J6/JFH-1-GFPΔ40 RNA, kindly provided by Jens Bukh (CO-HEP, Copenhagen), as described[1]. Replicons were selected using G418 (0.5 mg/ml) (Sigma) for ≥2 weeks. No G418 was used under experimental conditions.

Infectious supernatants were harvested from Huh7.5 cells 4 days post-electroporation, and titred by focus forming assay[1] (typically ~$1x10^5$ focus forming units (FFU)/ml) or using the Incuzyte Zoom (Cite Stewart et al., 2015 J virol methods). Huh7 cells were infected at an MOI of 0.5 and grown for 48 hr.

Wild-type Reovirus type 3 Dearing, Reolysin® (Reo, Oncolytics Biotech Inc.) was titred by L929 plaque assay. MV-Edm (Stephen Russell, Mayo Clinic, USA) was titred by Vero cell $TCID_{50}$ assay, as were Vaccinia and HSV-1.

Western blotting

HUH7-JFH1 cells were treated for 24 hr with the indicated treatments, then harvested and lysed in an equal volume of 2 x Laemmli buffer. For hepatocyte experiments, cells were lysed in EBC buffer (120

mM NaCl, 0.5% (v/v) Nonidet P-40, 50 mM Tris-Cl pH 8.0) supplemented with protease inhibitor cocktail (Roche) and protein concentration was determined by BCA assay[2]. Lysates were separated on 10 % SDS-polyacrylamide gels by electrophoresis and Western blotting was performed as previously described [1]. Antibodies used were sheep polyclonal anti-NS5A (1:5000), 1:10,000 anti-GAPDH (Sigma), 1:500 mouse-anti-Reo Sigma 3 (clone 4F2), 1:5000 mouseanti-βActin (Sigma) and appropriate horseradish-peroxidase conjugated secondary antibodies at 1:2500 (Sigma). Detection was by enhanced chemiluminescence using CL-XPosure Film (Thermo Scientific) and developed using a Konica SR0101 processor.

Isolation of PBMC using step-density gradient separation

Blood from healthy donors was diluted 2:1 with HBSS and layered onto Lymphoprep™, then centrifuged for 25 min with no brake. The white cell layer was isolated and washed twice with HBSS. PBMC were used immediately.

Reovirus replication and *UV*-irradiation

Cell lines and primary hepatocytes were incubated with Reo at 1 PFU/cell for 24 or 72 hours. Cells and supernatants were then collected and lysates generated by 3 cycles of freeze-thaw. Virus concentration was quantified using a standard L929 plaque assay.

Reo stocks were exposed to a range of durations of 254 nm UV light from an 8 watt bulb housed in a Stratalinker® UV Crosslinker 1800 (Stratagene). Inhibition of the viral lifecycle was determined by standard L929 plaque assay. The minimum duration of UV exposure necessary to abrogate productive infection was 2 minutes, and this was used to make *UV*-Reo.

MTT assays

Cells were treated for 72 hr for the assessment of cytotoxicity from reovirus infection, or as stated in other experiments. Following these treatment schedules, 3-[4,5-dimethylthiazol-2-yl]-2,5 diphenyl

tetrazolium bromide (Sigma) was added at 0.5 mg/ml. Plates were incubated for 4 hr, following which the media was removed and the cells solubilised using DMSO (Sigma). Optical density (OD) absorbance readings were determined using a Thermo Multiskan EX plate reader (Thermo scientific) at 550 nm absorbance.

Flow cytometry

Characterisation of immune cell populations in mixed liver cells and LMC; mixed liver cells or LMC were harvested, washed in FACS buffer and labelled with combinations of the antibodies described in table 1.

Table 1 Flow cytometry antibodies for immune cell characterisation

Target Molecule	Fluorochrome	Manufacturer
CD3	PerCP	BD Biosciences
CD4	FITC	BD Biosciences
CD8	FITC	BD Biosciences
γδ	PE	BD Biosciences
CD56	PE	AbD Serotec
CD19	PE	BD Biosciences
CD14	FITC	BD Biosciences
CD15	PerCP	BD Biosciences
CD16	FITC	BD Biosciences
Isotype	PerCP	BD Biosciences
Triple Isotype	PE, FITC, APC	Dako Cytomation

Assessment of cell viability by propidium iodide staining; Cell lines and primary hepatocytes were treated with either 1 PFU/cell Reo, 50 µM Z-VAD-FMK (R&D Systems) for 1 hr followed by 1 PFU/cell Reo, or PBS control followed by a 24 hr incubation. Adherent and suspension cells were harvested and stained with 0.05 mg/ml propidium iodide (PI; Sigma) and incubated at room temperature for 15 min. Data were acquired immediately.

Magnetic-activated cell sorting (MACS®) bead selection

PBMC NK cell depletions were performed using CD56 microbeads (Miltenyi-Biotec), according to the manufacturer's instructions.

Preparation of mixed liver cells, primary enriched hepatocytes and LMC

Ex-vivo normal liver and HCC samples were cultured in RPMI supplemented with 10 % FCS (Biosera) and 1 % (v/v) of Antibiotic Antimycotic Solution (Sigma). Liver tissue was passed through a 70 µm cell strainer (BD Biosciences), and the resultant single cell suspensions washed in HBSS. To obtain mixed liver cells, single cell suspensions were re-suspended in ACK buffer for 1 min, washed in cold HBSS and cultured.

To obtain LMC, the single cell suspensions were subjected to two rounds of density step-gradient centrifugation using Lymphoprep™ 800 x *g* for 25 min with no brake. Cells were collected from the interface layer and washed in HBSS prior to culture. To obtain enriched hepatocytes, the pelleted cells from the first density gradient centrifugation were were re-suspended in ACK buffer for 1 min, washed in cold HBSS and cultured. Plateable cryopreserved human hepatocytes (TRL/Lonza) were utilised in Fig 1c, d, e.

HCV plasmids

The subgenomic genotype 2a HCV plasmid, pRep-Feo-JFH1, was a kind gift from Professor Aleem Siddiqui. It carries a chimeric Neomycin Phosphotransferase (*NPT)/Firefly luciferase* (*Fluc*) gene (designated Feo) and has been previously described [3]. The full length (infectious HCV) genotype 2a J6/JFH1 recombinant plasmids, p2a(J6)-RLucΔ40 and p2a(J6)-GFPΔ40, have been described previously and was a kind gift from Professor Jens Bukh [4]. This plasmid encodes a chimeric virus where the core to NS2 genes are derived from the J6 genotype 2a sequence and the NS3 to NS5B genes are those of the genotype 2a JFH1 virus, with the additional insertion of the *Renilla luciferase* gene. Both plasmids carry ampicillin resistance genes.

Figure legends

Fig.S1. Replication and cytotoxicity of Reo in normal hepatocytes and HCC cell lines. **(A)** *Left:* Light microscope images of fractionated enriched hepatocytes and LMC. *Right:* Flow cytometry characterisation of immune cell populations in mixed liver cells and LMC. **(B)** Reo retrieval by plaque assay following 24 hr and 72 hr incubation with enriched hepatocytes (left) or HCC cell lines (right). **(C)** L929 plaque assay showing fold-change in concentration of input PFU following 24 hr or 72 hr incubation of Reo (1 PFU/cell) with HCC cell lines. **(D)** MTT assay of HCC cell lines incubated for 72 hr with 1 PFU/cell Reo. **(E)** Representative plots of propidium iodide killing assay using HCC cell lines incubated for 72 hr with PBS, 1 PFU/cell Reo or 1 PFU/cell Reo and the pan-caspase inhibitor Z-VAD-FMK.

Fig. S2. Reo-induced toxicity in SCID mice. **(A)** SCID mouse animal weight 20 days following a single Itu injection of PBS or $1x10^6$ PFU Reo into HUH7 or HUH7-JFH1 subcutaneous xenografts. **(B)** Plaque assay showing stained L929 cell monolayer following prolonged incubation (7 days) with 5000 PFU of UV-irradiated Reo. Time indicates duration of exposure to UV-irradiation.

Fig. S3. NK cells are necessary for Reo-stimulated PBMC-mediated killing of virus positive and negative HCC cell lines. **(A)** ^{51}Cr release assay using healthy donor PBMC pre-treated with PBS or 1 PFU/cell Reo for 24 hr. Replicate cells were depleted of NK cells, and PBMC were then co-incubated with ^{51}Cr-labelled HUH7 or HUH7-JFH1 targets for 4 hr. Data are ^{51}Cr release as a percentage of the potential maximum.

Fig. S4. Purified leukocyte-derived IFN-β potently inhibits HCV *in vitro*. **(A)** Luciferase assay (left) using HUH7-JFH1 cells treated for 24 hr with a range of concentrations and combinations of purified IFN-α, IFN-β and IL-29. Trendline equation (centre) for IFN-β derived from the luciferase assay. MTT assay (right) using replicate HUH7-JFH1 cells treated with purified IFN under the same conditions used in the luciferase assay. **(B)** MTT assay using HUH7-JFH1 cells under the same conditions used in the luciferase assay in Fig. 4 B. **(C)** Western blot for HCV NS5A and GAPDH using HUH7-JFH1 cells treated for 24 hr with a range of dilutions of RCM or CM at a dilution of 1:16 derived from JHH1, HLE or JHH2 cells, or with 20 µM 2'-C-Methylcytidine. **(D)** MTT assay using HUH7-JFH1 cells under the same conditions used in the luciferase assay in Fig. 4 D. **(E)** MTT assay using HUH7-JFH1 cells under the same conditions used in the luciferase assay in Fig. 4 F.

Fig. S5. Reo-conditioned media potently inhibit infectious HCV *in vitro*. Huh7 cells were infected with J6/JFH-1 chimeric HCV expressing an NS5A-eGFP reporter gene at an MOI of 1, followed by culture for 7 days to ensure the population was >98% infected with HCV. Cells were then exposed to either filtered control conditioned media (CM) or Reo-conditioned media (RCM) generated by exposure of mixed hepatic cells, or the JHH-1 or HLE cell lines to Reo. 24 hr post-treatment, cells were analysed for depreciation in HCV replication/gene expression **(A)** qRT-PCR for HCV RNA extracted from Huh7 cells using Trizol. **(B)** Comparison of NS5A-GFP positive cells following treatment with CM or RCM from mixed liver cells, JHH-1, or HLE sources. **(C)** Quantitation of NS5A-GFP cells corresponding to images in B, determined using the Incucyte Zoom.

Fig. S6. Reo exerts antiviral effects *in vivo.* **(A)** Representative IHC staining (brown) for HCV NS5A in HUH7-JFH1 subcutaneous xenografts in SCID mice treated with a single Itu injection of $1x10^6$ PFU Reo. **(B)** Luciferase (left) and MTT (right) assays for HUH7-JFH1 cells treated for 24 hr using CM or UV-Reo conditioned media derived from SCID mouse mixed liver cells stimulated with PBS or 1 PFU/cell UV-Reo.

Fig. S7. Other oncolytic viruses stimulate variable IFN responses. **(A)** MTT assay and ELISA for HBsAg secreted from PLC/PRF/5 cells treated for 5 days using purified IFN-β. **(B)** MTT assay for PLC/PRF/5 cells treated identically to those in Fig. 6 A. **(C)** ELISA for IFN-α, IFN-β, IFN-γ and IL-28B / IL-29 derived from mixed liver cells stimulated with various OV.

References

1 Griffin S, Stgelais C, Owsianka AM, *et al.* Genotype-dependent sensitivity of hepatitis C virus to inhibitors of the p7 ion channel. *Hepatology* 2008;**48**:1779–90. doi:10.1002/hep.22555

2 Smith PK, Krohn RI, Hermanson GT, *et al.* Measurement of protein using bicinchoninic acid. *Anal Biochem* 1985;**150**:76–85.http://www.ncbi.nlm.nih.gov/pubmed/3843705 (accessed 25 Sep2016).

3 Yokota T, Sakamoto N, Enomoto N, *et al.* Inhibition of intracellular hepatitis C virus replication by synthetic and vector-derived small interfering RNAs. *EMBO Rep* 2003;**4**:602–8.

4 Gottwein JM, Jensen TB, Mathiesen CK, *et al.* Development and application of hepatitis C reporter viruses with genotype 1 to 7 core-nonstructural protein 2 (NS2) expressing fluorescent proteins or luciferase in modified JFH1 NS5A. *J Virol* 2011;**85**:8913–28. doi:10.1128/JVI.00049-11

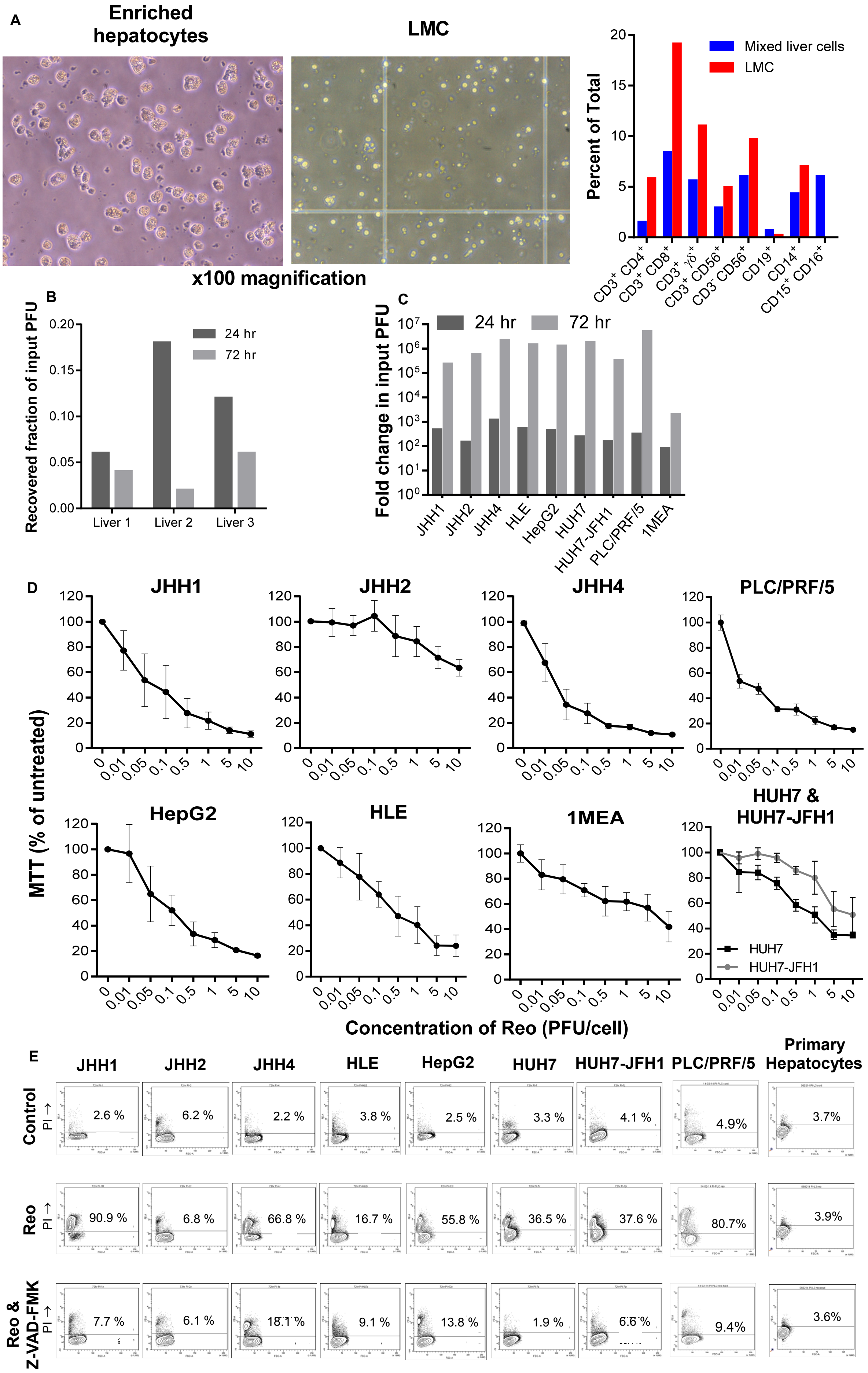

A
Enriched hepatocytes
LMC
x100 magnification
Mixed liver cells
LMC
Percent of Total
CD3+ CD4+
CD3+ CD8+
CD3+ γδ+
CD3+ CD56+
CD3- CD56+
CD19+
CD14+
CD15+ CD16+
B
Recovered fraction of input PFU
24 hr
72 hr
Liver 1
Liver 2
Liver 3
C
Fold change in input PFU
24 hr
72 hr
JHH1
JHH2
JHH4
HLE
HepG2
HUH7
HUH7-JFH1
PLC/PRF/5
1MEA
D
JHH1
JHH2
JHH4
PLC/PRF/5
HepG2
HLE
1MEA
HUH7 & HUH7-JFH1
HUH7
HUH7-JFH1
MTT (% of untreated)
Concentration of Reo (PFU/cell)
E
JHH1
JHH2
JHH4
HLE
HepG2
HUH7
HUH7-JFH1
PLC/PRF/5
Primary Hepatocytes
Control
PI →
2.6 %
6.2 %
2.2 %
3.8 %
2.5 %
3.3 %
4.1 %
4.9%
3.7%
Reo
PI →
90.9 %
6.8 %
66.8 %
16.7 %
55.8 %
36.5 %
37.6 %
80.7%
3.9%
Reo & Z-VAD-FMK
PI →
7.7 %
6.1 %
18.1 %
9.1 %
13.8 %
1.9 %
6.6 %
9.4%
3.6%

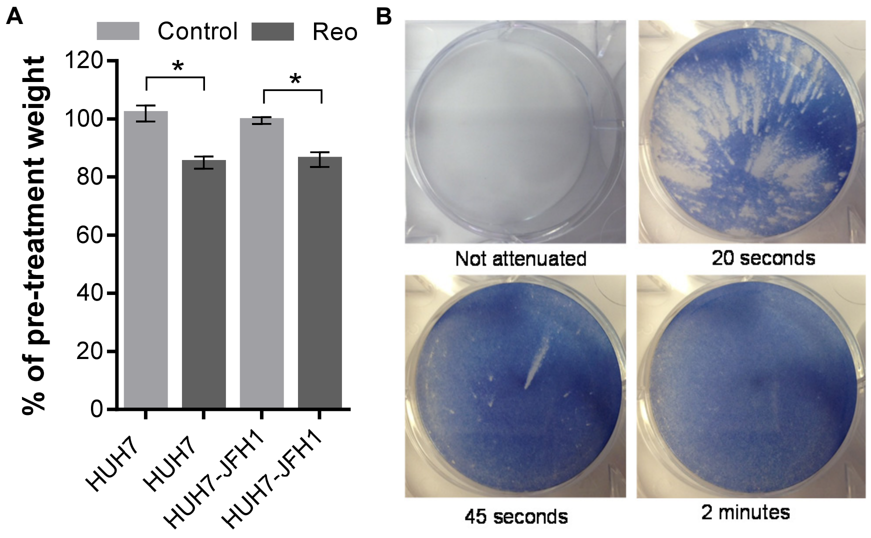
A
Control
Reo
% of pre-treatment weight
120
100
80
60
40
20
0
*
*
HUH7
HUH7
HUH7-JFH1
HUH7-JFH1
B
Not attenuated
20 seconds
45 seconds
2 minutes

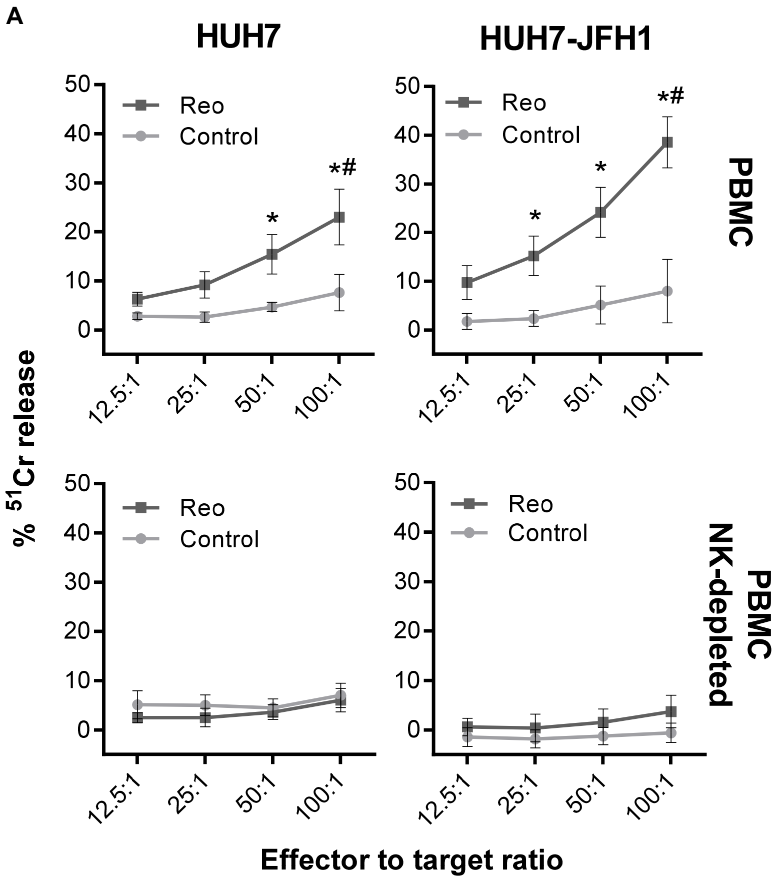

A
HUH7
HUH7-JFH1
PBMC
PBMC NK-depleted
Reo
Control
% ^{51}Cr release
*
*#
12.5:1
25:1
50:1
100:1
Effector to target ratio

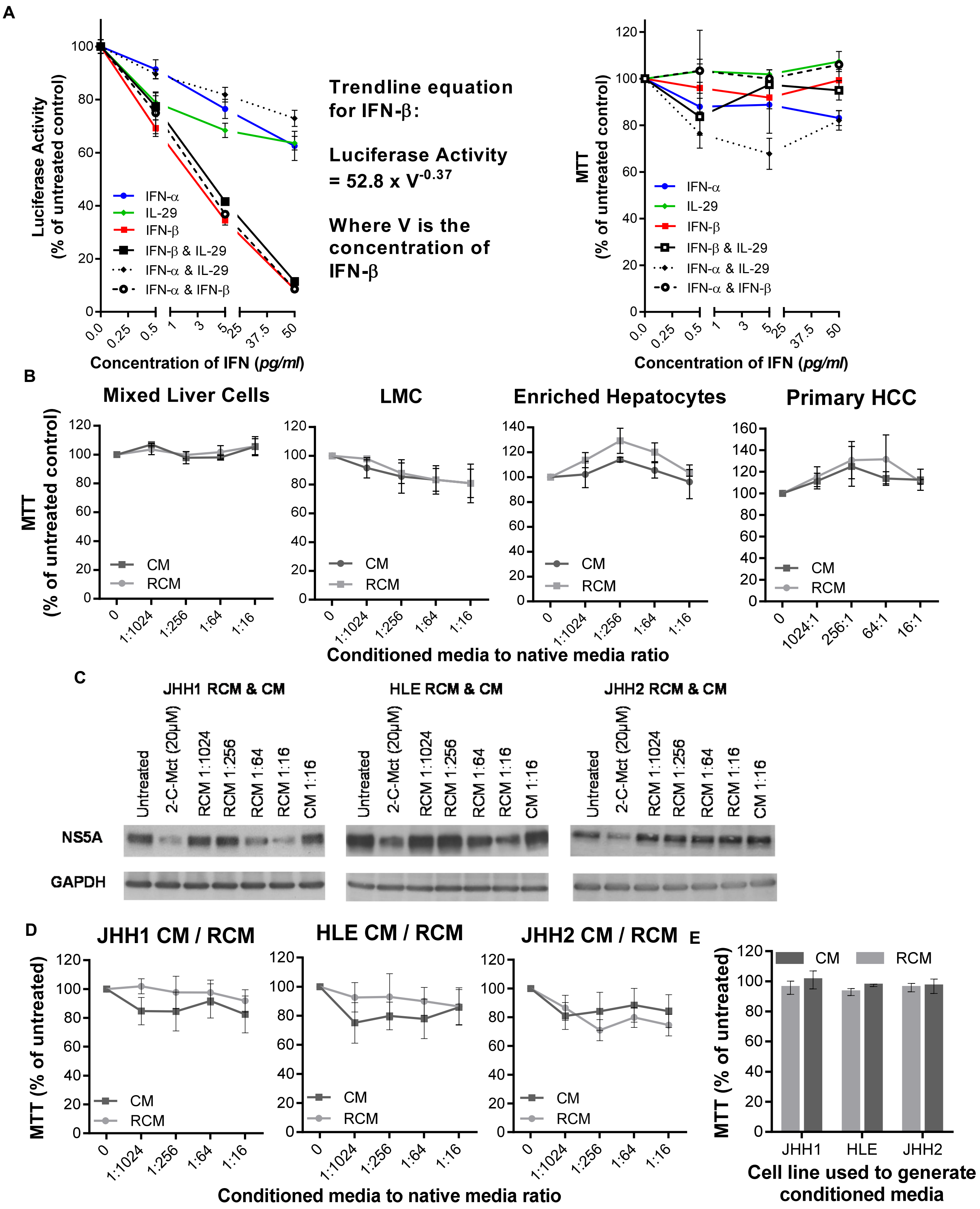
A
Luciferase Activity (% of untreated control)
IFN-α
IL-29
IFN-β
IFN-β & IL-29
IFN-α & IL-29
IFN-α & IFN-β
Concentration of IFN (pg/ml)
Trendline equation for IFN-β:
Luciferase Activity = 52.8 x V^-0.37
Where V is the concentration of IFN-β
MTT (% of untreated control)
B
Mixed Liver Cells
LMC
Enriched Hepatocytes
Primary HCC
CM
RCM
Conditioned media to native media ratio
C
JHH1 RCM & CM
HLE RCM & CM
JHH2 RCM & CM
Untreated
2-C-Mct (20µM)
RCM 1:1024
RCM 1:256
RCM 1:64
RCM 1:16
CM 1:16
NS5A
GAPDH
D
JHH1 CM / RCM
HLE CM / RCM
JHH2 CM / RCM
MTT (% of untreated)
Conditioned media to native media ratio
E
JHH1
HLE
JHH2
Cell line used to generate conditioned media

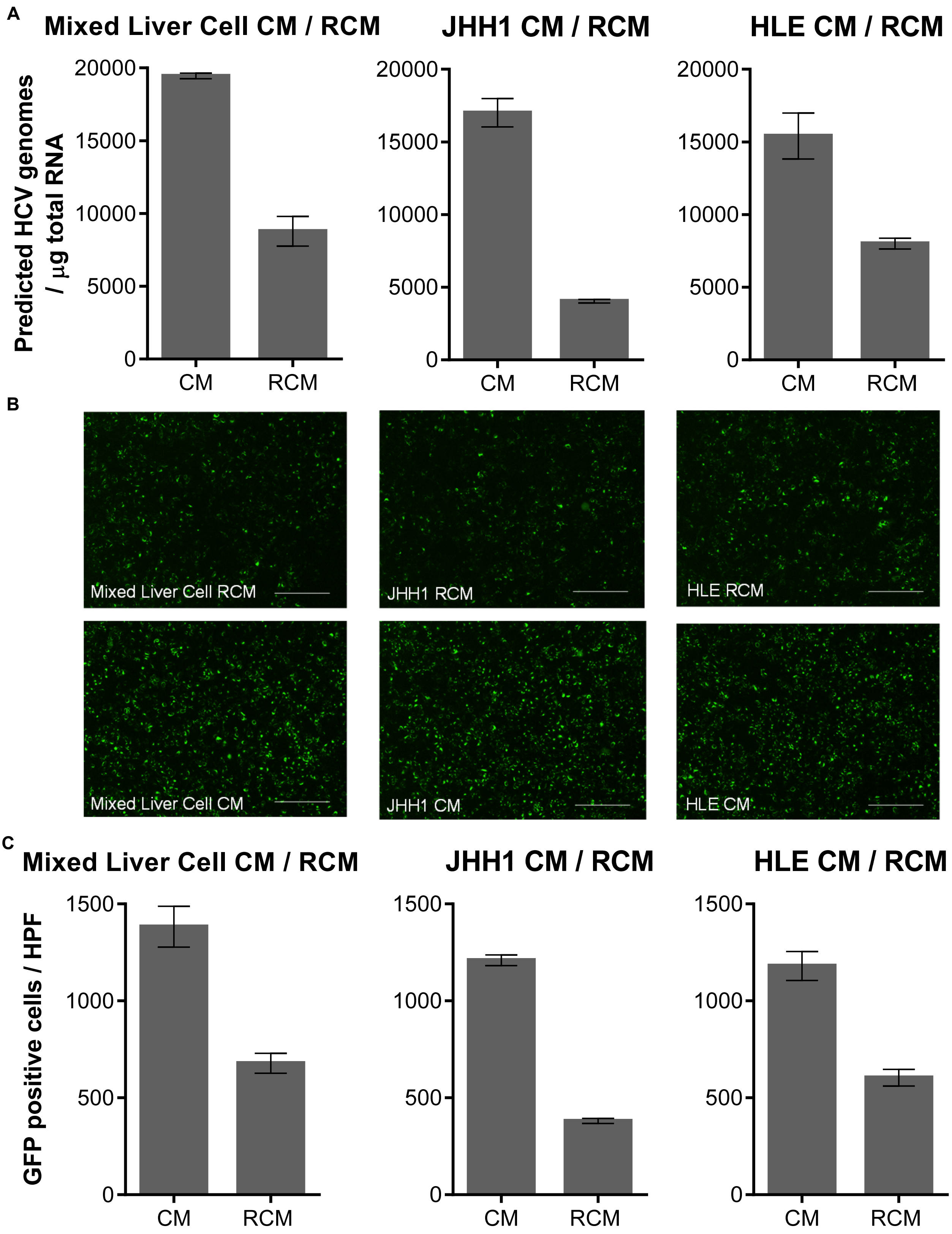

A
Mixed Liver Cell CM / RCM
JHH1 CM / RCM
HLE CM / RCM
Predicted HCV genomes / µg total RNA
20000
15000
10000
5000
0
CM
RCM
B
Mixed Liver Cell RCM
JHH1 RCM
HLE RCM
Mixed Liver Cell CM
JHH1 CM
HLE CM
C
Mixed Liver Cell CM / RCM
JHH1 CM / RCM
HLE CM / RCM
GFP positive cells / HPF
1500
1000
500
0
CM
RCM

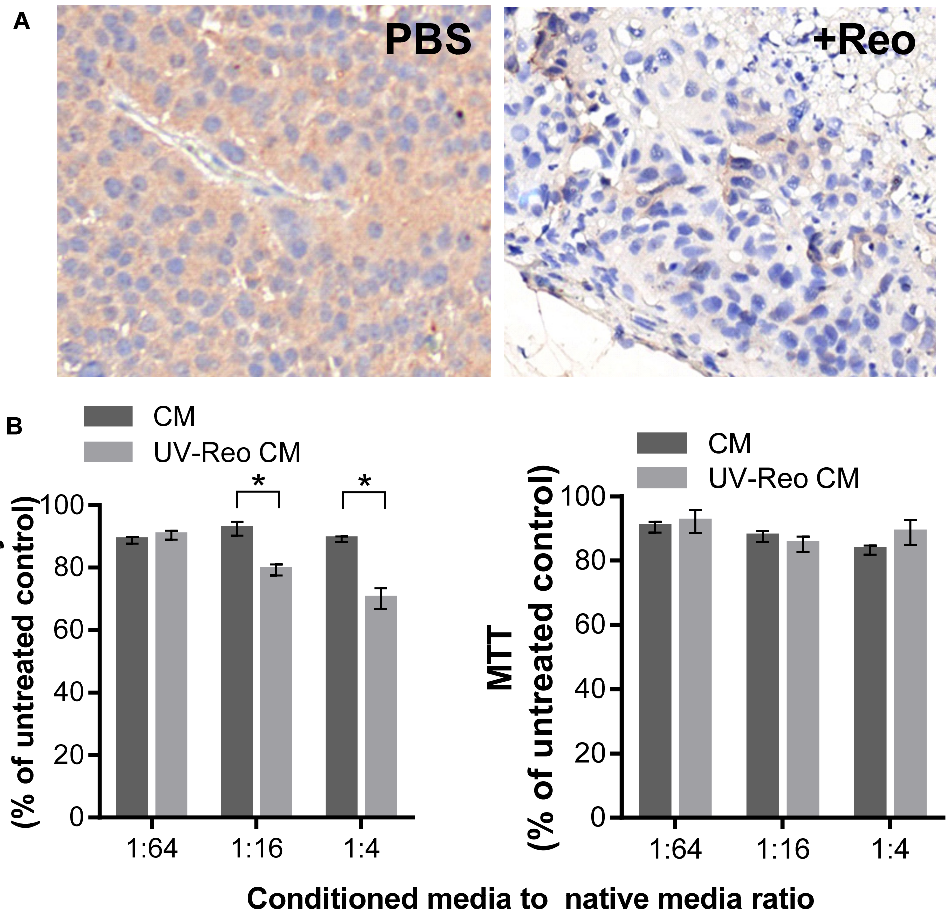

A
PBS
+Reo
B
CM
UV-Reo CM
Luciferase Activity
(% of untreated control)
100
80
60
40
20
0
*
*
1:64
1:16
1:4
MTT
(% of untreated control)
Conditioned media to native media ratio

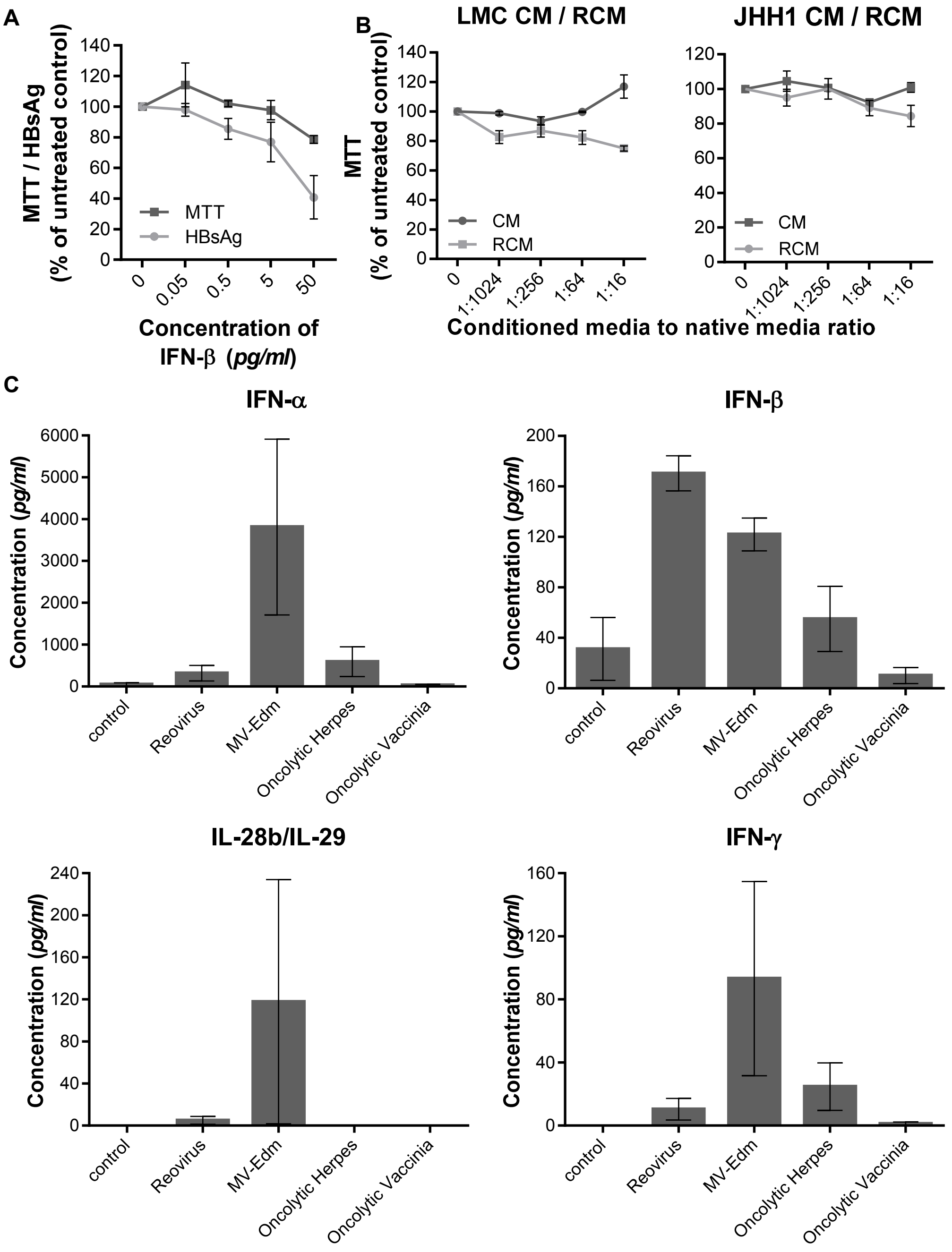

A
MTT / HBsAg (% of untreated control)
140
120
100
80
60
40
20
0
MTT
HBsAg
0
0.05
0.5
5
50
Concentration of IFN-β (pg/ml)
B
LMC CM / RCM
MTT (% of untreated control)
CM
RCM
0
1:1024
1:256
1:64
1:16
JHH1 CM / RCM
Conditioned media to native media ratio
C
IFN-α
Concentration (pg/ml)
6000
5000
4000
3000
2000
1000
control
Reovirus
MV-Edm
Oncolytic Herpes
Oncolytic Vaccinia
IFN-β
200
160
IL-28b/IL-29
240
IFN-γ
